# *Bacillus cabrialesii* subsp. *cabrialesii* Strain TE5: A Promising Biological Control Bacterium Against the Causal Agent of Spot Blotch in Wheat

**DOI:** 10.3390/plants14020209

**Published:** 2025-01-13

**Authors:** Ixchel Campos-Avelar, Michelle Fabiela García Jaime, Pamela Helué Morales Sandoval, Fannie Isela Parra-Cota, Sergio de los Santos Villalobos

**Affiliations:** 1 Instituto Tecnológico de Sonora, 5 de Febrero 818, Col. Centro, Cd. Obregón 85000, Mexico; ixchel_campos_avelar@hotmail.com (I.C.-A.); pamesandov37@gmail.com (P.H.M.S.); 2 Campo Experimental Norman E. Borlaug-INIFAP, Norman E. Borlaug Km. 12, Cd. Obregón 85000, Mexico; fipc04@gmail.com

**Keywords:** *Bacillus cabrialesii*, biocontrol, taxonomy, genome mining, sustainable agriculture

## Abstract

Strain TE5 was isolated from a wheat (*Triticum turgidum* L. subsp. *durum*) rhizosphere grown in a commercial field of wheat in the Yaqui Valley in Mexico. In this work, we present strain TE5 as a promising biological control agent against *Bipolaris sorokiniana*. First, after its genome sequencing through Illumina NovaSeq, this strain showed a genome size of 4,262,927 bp, with a 43.74% G + C content, an N50 value of 397,059 bp, an L50 value of 4 bp, and 41 contigs (>500 bp). Taxonomical affiliation was carried out by using overall genome relatedness indexes (OGRIs) and the construction of a phylogenomic tree based on the whole genome. The results indicated that strain TE5 identifies with *Bacillus cabrialesii* subsp. *cabrialesii*. Genomic annotation using Rapid Annotation Using Subsystems Technology (RAST) and Rapid Prokaryotic Genome Annotation (Prokka) indicated the presence of 4615 coding DNA sequences (CDSs) distributed across 330 subsystems, which included gene families associated with biocontrol, stress response, and iron competition. Furthermore, when the antiSMASH 7.1 platform was used for genome mining, the results indicated the presence of seven putative biosynthetic gene clusters related to the production of biocontrol metabolites, namely subtilosin A, bacillibactin, fengycin, bacillaene, bacilysin, surfactin, and rhizocticin A. Moreover, the antifungal activity of strain TE5 and its cell-free extract (CFE) was evaluated against *Bipolaris sorokiniana*, an emergent wheat pathogen. The results of in vitro dual confrontation showed fungal growth inhibition of 67% by strain TE5. Additionally, its CFE almost completely inhibited (93%) the growth of the studied phytopathogenic fungus on liquid media. Further observations of the impact of these bacterial metabolites on fungal spore germination exhibited inhibition of fungal spores through degrading the germinative hypha, avoiding mycelium development. Finally, the protective effect of strain TE5 against *Bipolaris sorokiniana* was evaluated for wheat seedlings. The results showed a significant decrease (83%) in disease severity in comparison with the plant infection without inoculation of the biological control agent. Thus, this work proposes *Bacillus cabrialesii* subsp. *cabrialesii* strain TE5 as a promising biological control agent against the wheat pathogen *Bipolaris sorokiniana* while suggesting lipopeptides as the potential mode of action, together with plant growth and defense stimulation.

## 1. Introduction

The world population is continuously increasing and is estimated to reach about 10 billion people by 2050 [1]. Thus, food production should increase to satisfy the predicted demand. Agriculture is an important activity which plays a vital role, as 80% of the consumed foods are produced through this sector [2]. One of the biggest challenges for agriculture is ensuring food security, especially during the adversities linked to climate change [3,4].

Furthermore, cereal crops are one of the most important foods, as they constitute the main source of nutrients around the globe. Worldwide, about 60% of the energy obtained from food is given by rice, maize, and wheat, which makes them basic foods for more than 7 billion people [5]. Thus, by 2050, cereal production should increase by a billion tons per year, where wheat is one of the most important cereals due to its energy and protein input [6]. Furthermore, the wheat agricultural surface is the biggest, and its commerce is superior in comparison with other cereal crops [7]. It is estimated that yearly production will reach approximately 775 million tons [8]. In Mexico, the annual production exceeds 3 million tons, with Sonora, Baja California, and Guanajuato being the main producers [7,9].

Agricultural production faces many threats, with climate change being one of the most challenging, as it alters plant physiology and the interactions between hosts, microorganisms, and the environment. Furthermore, it increases the incidence of pests and plant diseases, which cause about 30% of yield losses each year [10]. Among fungal phytopathogens, *Bipolaris sorokiniana* represents a major threat to wheat cultivars, as it is capable of infecting all wheat parts, including seeds, roots, shoots, and leaves, causing black point, root and crown rot, and spot blotch [11]. These diseases cause wheat yield losses ranging from 15 to 100%, depending on the region and the wheat genotype susceptibility [12]. To counteract biotic threats and increase crop production, synthetic pesticides and fertilizers are applied to agricultural fields under conventional practices [13]. However, these chemical additives represent a major risk to human health due to their nontarget toxicity as well as the environment, as the fraction not used by the plant may remain in the soil and water reservoirs as pollutants [14]. Moreover, chemical pesticides cause an important loss in soil fertility and biodiversity, and some of them have been documented as generators of pest and pathogenic resistance [15].

For these reasons, the implementation of sustainable agricultural practices has become vital to ensure food safety for future generations. Ideally, these innovative practices must preserve the environment and biodiversity while conferring resilience to crops to face the adverse environmental conditions of climate change. A promising strategy is the use of microbial inoculants based on specific and thoroughly characterized beneficial microorganisms [16,17], which can promote plant growth and control plant pathogens [18]. Indeed, microbial communities are essential for the regulation of biogeochemical cycles and the improvement of soil’s structure. Furthermore, microorganisms interacting with crops improve their resilience to abiotic and biotic stressors, which increases agroecosystem productivity [19]. Among microorganisms of agricultural importance, the genus *Bacillus* has exhibited the capacity to produce a wide range of metabolites and bioactive compounds, which allows for several beneficial traits, both as a plant growth promoter and as a biocontrol agent [20]. Furthermore, *Bacillus* strains have exhibited great tolerance to extreme environmental conditions due to its spore-forming capacity, which facilitates its formulation and increases its shelf life. For these reasons, it is widely studied as a source of microbial inoculants [21,22].

To accelerate the development of biological alternatives, the use of bioinformatics tools has become essential, as they facilitate the bioprospection of beneficial microbes with promising traits as effective microbial inoculants [23,24]. The process starts by ensuring a correct taxonomic affiliation [25,26], which allows the identification of promising species and the exclusion of those potentially harmful to plants and mammals, such as *Enterobacter cloacae* and *Burkholderia cepacia,* which are often studied as microbial inoculants despite possessing pathogenic traits [27]. Furthermore, the implementation of this strategy contributes to the search for putative bioactive compounds and the prediction of interactions between beneficial microorganisms and the host plant, as well as the deciphering of antagonistic mechanisms against plant pathogens [28].

The present study aims to contribute to the development of biological alternatives for achieving sustainable agriculture. For this, a native *Bacillus* strain (TE5) was isolated from the Yaqui Valley in Mexico, and bioprospecting against *Bipolaris sorokiniana*, an emergent wheat pathogen, through metabolic assays, genome sequencing, and mining for taxonomic affiliation was carried out, allowing the deciphering of potential action modes.

## 2. Results and Discussion

### 2.1. Morphological Characterization and Genomic Analysis of Strain TE5

Strain TE5 grown on nutrient agar presented off-white round and irregular colonies with a shiny texture. No swarming was observed, and microscopical observation showed short Gram-positive *Bacillus*. The spore-forming capacity of the strain was also verified. The genome resulted in 41 contigs (≥500 bp) with a total length of 4,262,927 bp, a G + C content of 43.74%, an N50 value of 397,059 bp, and an L50 value of four. An absence of contamination was observed through CheckM. Moreover, plasmids were also not detected by PlasmidFinder 2.1, validating the genomic stability of strain TE5 [29,30].

**Figure 1:**
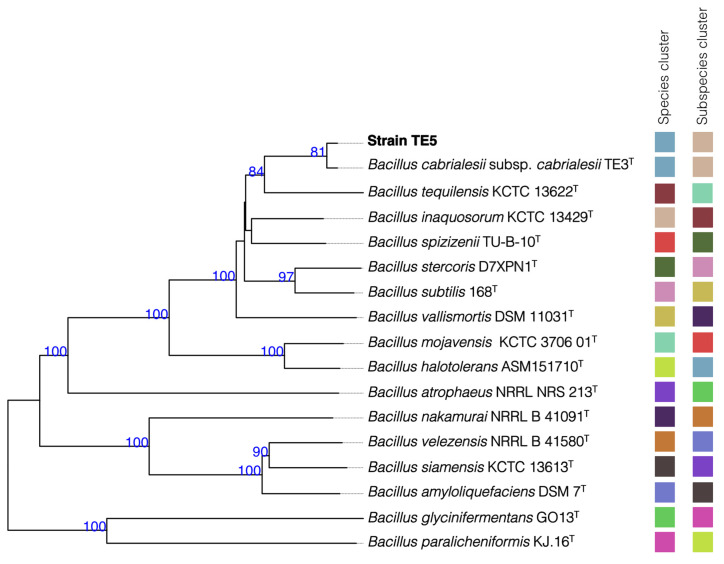
Phylogenetic relationship between strain TE5 and closely related type species based on genome sequences constructed using TYGS. Tree inferred with FastME 2.1.6.1 from GBDP distances calculated from genome sequences. The branch lengths are scaled in terms of the GBDP distance formula. The numbers above the branches are GBDP pseudo-bootstrap support values > 60% from 100 replications, with an average branch support of 73.0%.

Analysis of the 16S rRNA gene indicated that strain TE5 showed 100% similarity with both *Bacillus cabrialesii* subsp. *cabrialesii* TE3^T^ and *Bacillus inaquosorum* KCTC 13622^T^, while slightly lower similarity values were found with other species of the genus *Bacillus* (Table 1). However, according to the overall genome relatedness indexes (OGRIs) analysis, strain TE5 was taxonomically affiliated with *Bacillus cabrialesii* subsp. *cabrialesii* TE3^T^, as the obtained values were above the limits for species affiliation (ANI ≥ 95–96% and GGDC ≥ 70%) [31,32,33]. This taxonomic affiliation was confirmed by the generation of a phylogenomic tree with the type strain-related genomes (Figure 1), which support the phylogenomic similarity between strains TE5 and TE3^T^. *Bacillus cabrialesii* subsp. *cabrialesii* TE3^T^ has promising biotechnological traits, which have already been reported recently, for both plant growth promotion and biocontrol [34]. For instance, the TE3^T^ antagonistic potential has been evaluated against wheat pathogens such as *Bipolaris sorokiniana* TPQ3 [35] and *Fusarium languescens* CE2 [29].

### 2.2. Genome Annotation of Bacillus cabrialesii subsp. cabrialesii TE5

**Figure 2:**
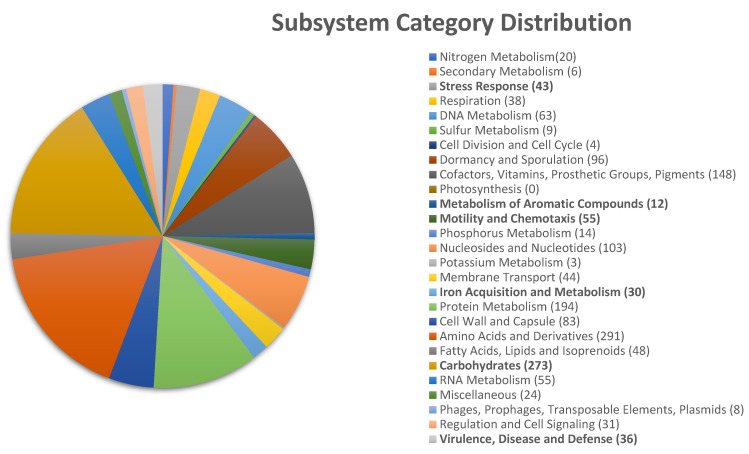
Subsystem category distribution of coding sequences from *Bacillus cabrialesii* subsp. *cabrialesii* TE5 constructed by RAST server version 2.0 CDSs.

The genome annotation performed by RAST indicated the presence of 4615 coding DNA sequences (CDSs) distributed across 330 subsystems (Figure 2). Notable subsystems included genes associated with stress response, mainly osmotic stress, and oxidative stress (43 CDSs). This represents an adaptive advantage because oxidative stress can result in damage at every sub-step of the central dogma, from hindering cellular replication to impeding protein synthesis or metabolic activity. Furthermore, it causes damage to both the backbone and bases of nucleic acids, whether free or incorporated, oxidizing amino acids and affecting protein cofactors [36]. On the other hand, RAST indicated the presence of gene clusters involved in iron competition by siderophore production (30 CDSs). Iron is an essential element for many plant metabolic processes, including nucleic acid synthesis, nitrogen fixation, and photosynthesis. It also serves as a cofactor in the synthesis of various enzymes, such as lipoxygenase and 1-aminocyclopropane-1-carboxylate oxidase [37]. Therefore, bacterial siderophores can promote the growth of the host plant by making iron ions available to plant roots [38]. Aside from that, bacteria capable of producing siderophores, as a survival mechanism, can promote plant growth by enhancing plant nutrition and limiting the development of pathogenic fungi and bacteria [39,40].

The RAST annotation revealed CDSs involved in the production of volatile organic compounds (VOCs) which stimulate plant growth and development [41], such as acetoin [18] and butanediol [42]. Also, CDSs involved in the metabolism of diverse carbohydrates like chitin, maltose, sucrose, lactose, and fructose (273 CDSs) were detected. Other notable subsystems included motility and chemotaxis (55 CDSs) as well as auxin biosynthesis (4 CDSs), such as the phytohormone indole-3-acetic acid (IAA), which has been shown to enhance plant growth-promoting effects through its involvement in processes such as cell division, elongation, tropism, apical dominance, senescence, flowering, and stress response [43,44]. RAST also revealed genes involved in virulence, disease, and defense mediated mainly by resistance to antibiotics like fluoroquinolones (*gyrA* and *gyrB*) and fosfomycin (*FosB*), as well as genes involved in the assimilation of toxic compounds such as chromium (*ChrA*), copper (*CIA*, *CopC*, and *CopD*), cobalt, zinc, and cadmium (*CzcD*), which were grouped into 36 CDSs.

### 2.3. Genome Mining of Bacillus cabrialesii subsp. cabrialesii TE5

Genome mining of *Bacillus cabrialesii* subsp. *cabrialesii* TE5 conducted with the web server AntiSMASH indicated the presence of five regions related to biosynthetic clusters of putative metabolites, such as subtilosin A (100% identity), bacillibactin (100% identity), fengycin (80% identity), bacillaene (100% identity), bacilysin (100% identity), surfactin (86% identity), and rhizocticin A (100%) (Table 2). The putative gene clusters found during genome mining analysis were similar to those previously reported for *Bacillus cabrialesii* subsp. *cabrialesii* TE3^T^ [45] and the recently affiliated *Bacillus cabrialesii* subsp. *cabrialesii* PE1 [29], which confirms the genomic stability of this clade. Surfactin is a cyclic lipopeptide which controls the infection of phytopathogens by directly attacking the membranes of phytopathogenic fungi or by inducing indirect systemic resistance in the host plant [46]. As a biosurfactant, surfactin also facilitates bacterial motility and attachment to diverse surfaces, contributing to the formation of persistent biofilms [47,48]. Another cyclic lipopeptide is fengycin, which has strong antifungal activity, specifically against filamentous fungi, by altering the structure and permeability of the fungal cell membrane [49]. These lipopeptides were suggested to be responsible for mediating the antifungal activity exhibited by the cell-free culture in the assays of the type strain of *Bacillus cabrialesii* subsp. *cabrialesii* TE3^T^ against the phytopathogen *Bipolaris sorokiniana* TPQ3, according to the results obtained through untargeted mass spectrometry (HPLC-ESI-MS/MS) [45]. The compound identified as rhizocticin A is a dipeptide with antifungal activity which penetrates fungal cells via the oligopeptide transport system. This penetration leads to the release of a non-protein phosphate-containing amino acid, which inhibits threonine synthesis [50]. Phosphonate compounds are prevalent among biologically active substances primarily due to their ability to affect carboxy- and phosphate-containing metabolites [51]. On the other hand, the siderophore bacillibactin is an iron chelator which has the potential to bind soluble iron ions, which are essential for the activity and growth of pathogens. Thus, if the siderophore produced by the antagonist can outcompete pathogenic siderophore production, then this can lead to the inhibition of pathogenic growth, hence indirectly conferring biocontrol activity, as has been previously reported for bacillibactin, for which direct antimicrobial activity was also suggested [52]. Meanwhile, subtilosin and bacilysin are compounds with important antimicrobial activity [53,54].

The abbreviations used in this context are as follows: non-ribosomal peptide synthetase (NRPS), polyketide synthetases (PKSs), type III PKS (T3PKS), Betalactone-containing protease inhibitor (Betalactone), non-ribosomal peptide metallophore (NRP-Metallophore), and “Other” (a category encompassing secondary metabolite-related proteins which did not fit into any other specific category).

### 2.4. Antagonistic Capacity of Bacillus cabrialesii subsp. cabrialesii TE5 Against Bipolaris sorokiniana

#### 2.4.1. Dual Confrontation Assay

**Figure 3:**
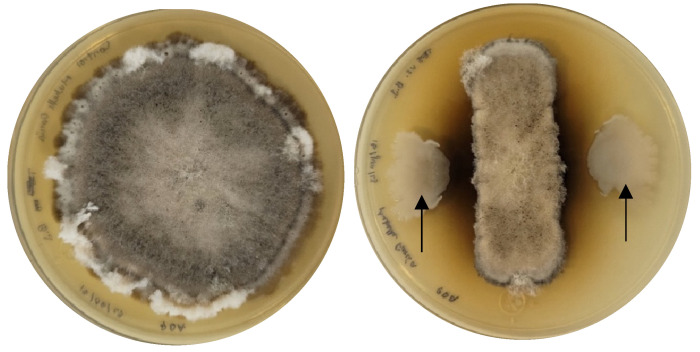
Dual confrontation of *Bipolaris sorokiniana* TPQ3 and bacterial strain TE5 on PDA plates after 7 days of culture at 30 °C. **Left**: Fungal inoculum alone. **Right**: Fungal inoculum in confrontation with strain TE5 (arrows).

The antagonistic capacity of *Bacillus cabrialesii* subsp. *cabrialesii* TE5 against *B. sorokiniana* on PDA plates showed a mean fungal growth inhibition of 67% (±7%). The observed antagonistic mechanism was diffusible metabolites (Figure 3), which is a common antagonistic mechanism reported among the genus *Bacillus* [55,56] and was also previously reported for this subspecies [34]. This strongly suggests that the actual synthesis of bioactive metabolites predicted by the AntiSMASH analysis could be related to the observed antifungal bioactivity. For example, the ability of the genus *Bacillus* to antagonize phytopathogens is strongly associated with the production of cyclic lipopeptides, such as surfactin and fengycin, together with the antifungal dipeptide rhizocticin A and the siderophore bacillibactin, which enables nutrient competition [57]. Moreover, while the polyketide bacillaene has been reported as having antifungal properties [58,59], subtilosin and bacilysin are antimicrobials with activity against closely related microbial members [60].

Interestingly, during dual confrontations against strain TE5, *B. sorokiniana* TPQ3 also produced diffusible pigments toward the areas where the bacteria produced a halo of fungal inhibition. These melanin-like pigments are a common response of fungi to stressing factors, and they act as a defense mechanism against oxidative stress, poor nutrient supply, and toxic compounds [61,62]. Further analysis by transcriptomic studies could help elucidate the interaction mechanisms occurring between both microorganisms during confrontation, as reported by recent studies on the antifungal mechanisms of *Bacillus amyloliquefaciens* against *Alternaria solani* [63] and *Fusarium oxysporum* [64], which evidenced an upregulation of antifungal compounds produced by the bacterial antagonist on the presence of the fungal pathogen.

#### 2.4.2. Impact of Diffusible Metabolites of *Bacillus cabrialesii* subsp. *cabrialesii* TE5 on *B. sorokiniana* Growth

**Figure 4:**
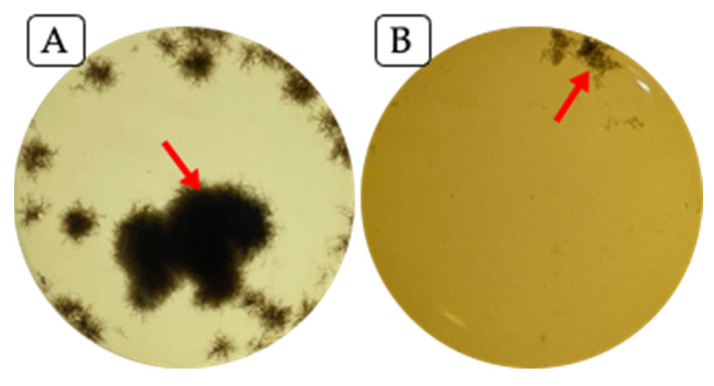
*Bipolaris sorokiniana* growth inhibition by cell-free extract of *Bacillus cabrialesii* subsp. *cabrialesii* TE5. The interaction experiment was conducted for 3 days at 30 °C and 120 rpm. (**A**) Fungal inoculum alone on PDB medium. (**B**) Fungal inoculum on PDB medium amended with 47% of CFE produced by strain TE5. Red arrows indicate fungal mycelium.

The cell-free extract (CFE) of strain TE5 was obtained after culturing the bacteria for 72 h on NB medium and filtering the supernatant through a polyethersulfone (PES) filter (0.22 µm). The results show that the bacterial CFE decreased the mycelial growth (area) of *B. sorokiniana* by 93% (Figure 4). A recent study reported inhibition of *B. sorokiniana* of almost 93% by *Bacillus amyloliquefaciens* through its diffusible metabolites, which could indicate a common antagonistic mechanism between this species and strain TE5 [20]. Recent genome mining of *B. amyloliquefaciens*, with antifungal activity against *Fusarium graminearum*, reported the presence of diverse putative gene clusters, some of which are common to those found for strain *Bacillus cabrialesii* subsp. *cabrialesii* TE5, namely surfactin, fengycin, and bacillibactin, suggesting their implication in the observed antifungal effect [65]. Furthermore, the observed fungal inhibition due to the CFE of *Bacillus cabrialesii* subsp. *cabrialesii* TE5 indicates that antifungal metabolites are biosynthesized naturally without the stimulus of the fungal cells’ presence. Moreover, further studies on the stimulation of bacterial metabolism should be performed to optimize culture media and increase the production of bioactive compounds for biocontrol applications [53].

#### 2.4.3. Impact of Bacterial CFE on Fungal Spore Germination

**Figure 5:**
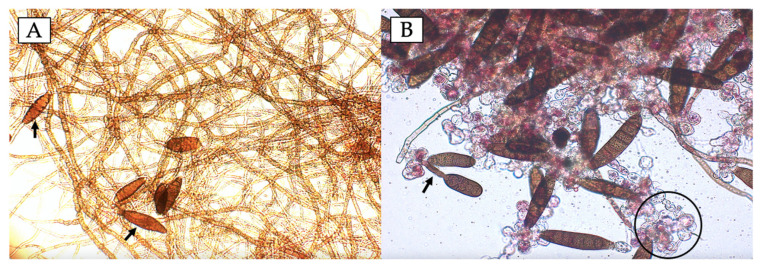
*Bipolaris sorokiniana* TPQ3 spores’ germination after incubation in PDB medium (**A**) or PDB medium containing 47% cell-free extract of *Bacillus cabrialesii* subsp. *cabrialesii* TE5 (**B**). Incubation was conducted for 24 h at 30 °C and 120 rpm. Tetrazolium red was added to assess cell viability. (**A**) Abundant fungal mycelia and few well-defined spores (arrows). (**B**) Spores exhibiting poorly developed germination tubes (arrow) and lysed spores with leaked cellular content (circle).

As observed in Figure 5, the treatment of fungal inoculum alone without bacterial CFE (Figure 5A) showed a large amount of long fungal mycelium and few ungerminated typical spores. On the other hand, the fungal spores which interacted with the CFE of strain TE5 (Figure 5B) showed a major spore disruption, with damaged cellular membranes and deficient germination. This type of disrupted fungal spore germination is highly associated with the bioactivity of lipopeptides, such as iturin, surfactin, and fengycin [66], of which the latter two were present in the putative gene clusters of the genome of strain TE5 (Table 2). Fungal spore germination inhibition has been linked to disruption of the lipopeptides of the fungal membrane, which alters its permeability and induces the leakage of nucleic acids and proteins, leading to cell lysis. Furthermore, changes in fungal metabolic processes and gene expression have been documented in the presence of surfactin, as well as its binding to DNA molecules, hindering replication and transcription mechanisms [67,68].

### 2.5. Biocontrol Capacity of Strain TE5 Against Bipolaris sorokiniana on Wheat Seedlings

**Figure 6:**
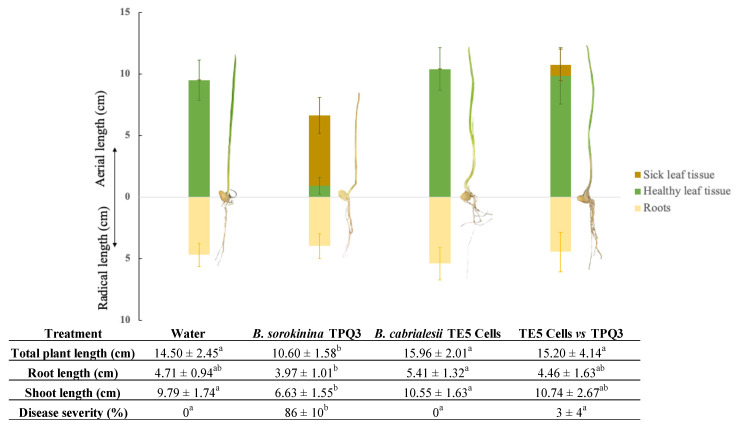
Biometric parameters of wheat plantlets during evaluation of the protective effect of *Bacillus cabrialesii* subsp. *cabrialesii* TE5 against wilting caused by *Bipolaris sorokiniana* TPQ3. The bar plot shows the mean and standard deviation, as well as representative seedlings for each treatment. Numeric results are presented in the bottom table. Disease severity was calculated as a percentage of wilting of the plantlets’ leaves. Results with different letters are significantly different according to the Kruskal–Wallis test and Wilcoxon post hoc treatment (*p* ≤ 0.05).

The capacity of strain TE5 to preserve plant health against infection with *Bipolaris sorokiniana* TPQ3 was evaluated by soaking the shoots of wheat seedlings in a bacterial suspension (1 × 10^4^ CFU/mL) before infecting them with the phytopathogen. The results show that the inoculation of *Bacillus cabrialesii* subsp. *cabrialesii* TE5 showed significant (*p* ≤ 0.05) protection of wheat seedlings against *B. sorokiniana* TPQ3, as signs of wilting decreased considerably (−83%) in comparison with those inoculated only with the phytopathogen (Figure 6).

The ability of *Bacillus cabrialesii* to prevent infection caused by *B. sorokiniana* has previously been reported for the strain TE3^T^, where the application of bacterial cells 24 h before fungal infection decreased the number of lesions from about 6.5 lesions/cm^2^ to roughly 3 lesions/cm^2^. Thus, the protective effect of this bacteria after foliar inoculation suggested its ability to colonize the wheat phyllosphere and remain viable to ensure plant protection [35]. As stated before, diverse compounds could be behind the antifungal effect observed by destroying fungal cell membranes or inducing plant systemic resistance, such as the siderophore bacillibactin, the dipeptide rhizocticin A, and the lipopeptides surfactin and fengycin, which also mediate root colonization processes [69].

Our results also show a slight increase in the plant biometric parameters (shoot length and root length) through inoculation with strain TE5 in comparison with those seedlings treated only with sterile water, which was used as a control (Figure 6). In this sense, a recent study showed the capacity of *B. cabrialesii* TE3^T^ to promote plant development, as its inoculation in wheat plants significantly increased their aerial lengths [70]. Furthermore, the association of *B. cabrialesii* with other beneficial plant microbes has exhibited a synergic effect on plant growth promotion, as reported by Rojas-Padilla et al. in 2022, where a consortium of *B. cabrialesii* TE3^T^ and *B. megaterium* (now *Priestia megaterium*) TRQ8 significantly increased the stem lengths (+11.7%), root lengths (+7.9%), and stem dry weights (+30.5%) of wheat plants [71]. This offers new possibilities for the development and study of native microbial consortia for managing wheat plant pathogens.

Overall, the obtained results reinforce the predicted biotechnological potential of *B. cabrialesii* subsp. *cabrialesii* TE5 for improving wheat resilience. Indisputably, *in planta* validation is critical to ensure the efficacy of microbial inoculants, as the complexity of interactions increases when a plant is included [21]. However, integrated omic approaches have allowed major advances in the bioprospection of numerous beneficial microorganisms both for biocontrol and biofertilization as well as in the elucidation of the involved mechanisms [45,72].

## 3. Materials and Methods

### 3.1. Bacterium Isolation and Culture Conditions

Bacterial isolation of strain TE5 (GenBank accession number: JBEJUL000000000) was performed following a previously published protocol [73]. Briefly, a mixed soil sample was composed of 6 different individual samples, all of which were collected from the rhizosphere of a commercial wheat field. This field is located in the Yaqui Valley in Sonora, Mexico ((27°35′53.14″ N and 110°2′53.26″ W), and it was subjected to conventional agricultural practices for at least 4 decades. A suspension of 10 g in 90 mL of sterile distilled water was used to perform 10 fold serial dilutions. The previous soil-water mixture was diluted by the serial dilution method (1:10) up to 10^−6^. One mL of each dilution was spread on a Petri dish containing nutrient agar (NA) in triplicate, and they were incubated at 28 °C for 2 days. One of the most abundant bacterial strains (TE5) was purified, and its morphology was characterized macro- and microscopically, describing its colony shape, color, elevation, and opacity as well as its Gram staining and spore-forming capacity by heating the bacterial suspension at 80 °C for 10 min before inoculation on NA plates. Furthermore, strain TE5 was cryopreserved at −80 °C with the addition of 30% glycerol at the Colección de Microorganismos Endófitos y Edáficos Nativos (COLMENA, http://apps2.itson.edu.mx/colmena/ accessed on 24 July 2024) [74].

### 3.2. Genome Sequencing and Assembly

High-quality bacterial genomic DNA of strain TE5 was extracted and analyzed following a previously published protocol [73]. Briefly, a fresh culture of strain TE5 was prepared by inoculating 1 × 10^4^ colony forming units (CFUs) on 20 mL of nutrient broth (NB) medium and incubation at 30 °C and 120 rpm for 24 h. After incubation, the bacterial cells were centrifuged for 5 min at 3630× *g*, and the pellet was used for DNA extraction as described by Raeder and Broda (1985) [75]. The genomic DNA of strain TE5 was sequenced using the Illumina NovaSeq platform, and Trimmomatic version 0.40 [76] (http://www.usadellab.org/cms/?page=trimmomatic accessed on 24 July 2024) was used to remove low-quality bases and adapter sequences. Moreover, the de novo assembly was carried out with SPAdes version 3.15.5 [77] using the “careful” parameter for error correction in reads. The assembled contigs were ordered with Mauve contig Mover version 2.4.0 [78] using the reference genome of *Bacillus cabrialesii* subsp. *cabrialesii* TE3^T^ (GenBank accession number: GCA_004124315.1) according to the highest similarity of the 16S rRNA gene (100% similarity and 100% completeness). Plasmid detection was performed using PlasmidFinder 2.1 [79], and contamination was detected using CheckM version 1.0.18 [30].

The obtained genome sequence was submitted to the EzBioCloud database to determine the most closely related strains, regarding the cutoff values for species delimitation established for the 16S rRNA gene (>98.7%) [80]. Furthermore, to taxonomically affiliate strain TE5, its genome was compared to the more closely related strains using the average nucleotide identity (ANI) through the OrthoAni algorithm [81], ANIb through the BLAST algorithm, ANIm using MUMmer, as well as Genome-to-Genome Distance Calculator (GGDC) version 2.1, which uses the BLAST algorithm [82]. Finally, a phylogenomic tree was created using the Type Strain Genome Server (TYGS) platform to explore the evolutionary relationship with other whole bacterial genomes related to strain TE5, and *Bacillus mexicanus* FSQ1 (GenBank accession number: GCA_020223575.2) was used as an outgroup [83].

#### Genome Annotation and Mining

The genome annotation of strain TE5 was performed using the Rapid Annotation Using Subsystems Technology (RAST) server version 2.0 (www.rast.nmpdr.org) (accessed on 15 February 2024) with the default RASTk pipeline [84,85]. Rapid Prokaryotic Genome Annotation (Prokka) was also used as a second annotation platform for assessing accuracy through the DOE Systems Biology Knowledgebase platform (KBase, http://kbase.us accessed on 24 July 2024) [86,87]. To identify the biosynthetic potential of strain TE5, its genome was submitted to the Antibiotics and Secondary Metabolite Analysis Shell (AntiSMASH) web server version 7.0 (www.antismash.secondarymetabolites.org) (accessed on 15 February 2024) under the “relaxed” parameter, which allowed the identification, annotation, and analysis of gene clusters related to the biosynthesis of secondary metabolites, non-ribosomal peptide synthetases, polyketide synthases, type I and II polyketide synthases, lasso peptides, and antibiotic oligosaccharides, among others [88].

### 3.3. The Antagonistic Capacity of Strain TE5 Against Bipolaris sorokiniana TPQ3

*Bipolaris sorokiniana* TPQ3, a phytopathogenic strain isolated from wheat fields by our research team [89] and belonging to COLMENA [74], was used in this assay. Thus, for fungal spore production, Petri dishes containing V8 medium (V8 juice: 200 mLL^−1^; CaCO_3_: 2 gL^−1^; agar: 15 gL^−1^) were inoculated with fresh fungal mycelium from a stored (4 °C) PDA plate of the fungus and incubated at 30 °C for 7 days. After the incubation period, fungal spores were collected by adding 5 mL of sterile distilled water with 0.01% Tween 80. The spores were scraped from the surface of the fungal colony and enumerated on a Neubauer chamber. The fungal spore suspension was then adjusted to 1 × 10^4^ spores/mL for further assays.

The dual confrontation assay was performed according to the protocol described by Montoya-Martínez et al. [28] with modifications. Briefly, 10 µL of a *B. sorokiniana* TPQ3 spore suspension was inoculated at the centers of Petri dishes containing potato dextrose agar (PDA). Furthermore, 10 µL (1 × 10^4^ CFU/mL) of strain TE5 was inoculated on each side of these Petri dishes 1 cm from the border. For the treatment with fungal inoculum alone, bacteria were not inoculated. Assays were performed in triplicate. The inoculated plates were incubated at 30 °C for 7 days. Fungal growth inhibition was measured after 7 days through image analysis with ImageJ software version 1.53. The fungal inoculum alone growth area was established as 100%, and the percentage of fungal inhibition due to bacteria inoculation was determined using the following equation:

$$Fungal\;growth\;inhibition\left(\%\right)=\left(1-\frac{Area\;of\;fungal\;growth\;in\;confrontation\;\left({cm}^{2}\right)}{Area\;of\;fungal\;growth\;alone\left({cm}^{2}\right)}\right)\times 100$$

#### 3.3.1. Cell-Free Extract (CFE) of Strain TE5 and Its Antagonistic Capacity Against *B. sorokiniana*

The antagonistic capacity of the cell-free extract (CFE) obtained from TE5 was determined according to previously published protocols [28]. Briefly, a bacterial pre-inoculum was prepared by inoculating a loop of a fresh colony of strain TE5 into a tube containing 20 mL of nutrient broth (NB) and culturing at 30 °C and 120 rpm for 24 h. After incubation, the bacterial suspension was centrifuged at 3630× *g* for 10 min, and the supernatant was discarded. The resulting pellet was washed twice with sterile distilled water and resuspended in 20 mL of sterile distilled water. The optical density (OD) at 630 nm was measured, and the bacterial cell suspension was adjusted to 0.5 = 1 × 10^4^ CFU/mL. For CFE production, 100 µL of the washed pre-inoculum suspension was added to a flask containing 20 mL of NB and incubated at 30 °C and 120 rpm for 72 h. Furthermore, the bacterial suspension was centrifuged at 14,530× *g* for 5 min, and the supernatant was filtered through a 0.22 µm polyethersulfone (PES) filter. The absence of bacteria was confirmed by inoculating 1 mL of the CFE on Petri dishes containing NA. The antifungal capacity of the bacterial CFE was evaluated against *B. sorokiniana* TPQ3 on 24 well microplates. The experimental design (*n* = 12) consisted of (1) 750 µL of bacterial CFE, 750 µL of potato dextrose broth (PDB), and 100 µL (1 × 10^4^ spores/mL) of the fungal spore suspension and (2) 750 µL of sterile distilled water, 750 µL of potato dextrose broth (PDB), and 100 µL (1 × 10^4^ spores/mL) of the fungal spore suspension (treatment with fungal inoculum alone). Microplates were sealed and incubated at 30 °C and 120 rpm for 72 h. Moreover, high-quality photographs were taken to quantify the fungal inhibition through image analysis using ImageJ according to the formula described previously. The impact of the TE5 CFE on fungal spore germination was observed with optical microscopy. For this, the conditions of the previous assay were replicated and incubated for 24 h, with the addition of 10% tetrazolium red to the culture to facilitate observation of the integrity of the fungal cells.

### 3.4. Evaluation of Biocontrol Capacity of Strain TE5 Against Bipolaris sorokiniana on Wheat Seedlings

The wheat seeds (CIRNO 2008) were surface disinfected by submerging them in an ethanol solution (70% *v*/*v*) for 2 min and then a hypochlorite solution (2% *v*/*v*) for 2 min before finally rinsing them trice with sterile water. The disinfected seeds were then germinated in a plastic container with a wet sterile paper towel for about 3 days and until the shoots measured about 1 cm long.

For the inoculation of these seedlings, the shoots were submerged in the following four treatments: (1) sterile distilled water; (2) *Bipolaris sorokiniana* TPQ3 spore suspension (1 × 10^4^ spores/mL); (3) bacterial cells of *Bacillus cabrialesii* subsp. *cabrialesii* TE5 produced as described in Section 3.3.1; and (4) *Bacillus cabrialesii* subsp. *cabrialesii* TE5 versus *Bipolaris sorokiniana* TPQ3. For treatment (4), the shoots were submerged in the bacterial treatment first and then dried under a sterile laminar flow before being successively submerged in the fungal spore suspension.

Once inoculated and dried, the seedlings were placed in glass tubes with bacteriological agar at 1.5% and left to grow at 26 °C and 45% RH for 7 days and a photoperiod of 12 h of light and 12 h of darkness. After the incubation period, the plantlets were collected from the tubes and photographed before measuring the following three biometric parameters: shoot length, radical length, and wilt (brownish leaf tissue) length. The results were plotted as the mean of at least 6 replicates, and statistical analyses were performed using RStudio software (2024.04.2+764). Disease severity was calculated using the following equation:

$$Disease\;severity\;\left(\%\right)=\left(\frac{Wilt\;length\;\left(cm\right)}{Shoot\;length\;\left(cm\right)}\right)\times 100$$

The normal distribution of data was verified with the Shapiro–Wilk normality test. Statistical analysis of non-parametric data was conducted using the Kruskal–Wallis ANOVA on ranks. When significant differences were found (*p* ≤ 0.05), pairwise comparisons were performed using the post hoc Wilcoxon method with a 95% confidence interval.

## 4. Conclusions

As agriculture faces increased challenges linked to the control of crop diseases, the development of sustainable strategies for the improvement of crop resilience and yield has become a main focus for the scientific community. The results presented in this work allowed us to affiliate strain TE5 with *Bacillus cabrialesii* subsp. *cabrialesii*. Furthermore, strain TE5 exhibited great antagonist activity against *Bipolaris sorokiniana* in vitro both by confrontation and by diffusible metabolites, which seemed to be biosynthesized in the absence of the studied phytopathogenic fungus. Based on genome mining and microscopic observation of the fungal spores, the potential metabolites involved in this bioactivity are surfactin and fengycin. Finally, the biotechnological potential of strain TE5 was confirmed in vivo, as inoculation of this strain effectively protected wheat seedlings against *B. sorokiniana* infection. Thus, our study reports *Bacillus cabrialesii* subsp. *cabrialesii* TE5 as a promising alternative for the development of microbial inoculants for the biocontrol of plant pathogens on wheat crops, either by the application of bacterial cells or their metabolites. Further studies on the elicitation of bacterial secondary metabolism could also be implemented to optimize production of the identified antifungal compounds and their evaluation under field conditions.

## Figures and Tables

**Table 1:** The 16S rRNA gene and OGRI-based taxonomic affiliation of strain TE5.

Taxon Name	Strain	Accession Number	Similarity (%)	Ortho ANI Value (%)	GGDC Formula 2 (%)
*Bacillus cabrialesii* subsp. *cabrialesii*	TE3 (TYPE)	GCA_004124315.1	100	99.23	93.6
*Bacillus inaquosorum*	KCTC 13429 (TYPE)	GCA_003148415.1	100	93.82	54.2
*Bacillus tequilensis*	KCTC 13622 (TYPE)	GCA_000507145.1	99.86	93.67	53
*Bacillus stercoris*	D7XPN1 (TYPE)	GCA_000738015.1	99.86	92.21	46.6
*Bacillus spizizenii*	TU-B-10 (TYPE)	GCA_000227465.1	99.86	93.74	53.6
*Bacillus subtilis*	168 (TYPE)	GCA_000009045.1	99.79	92.36	47.7
*Bacillus halotolerans*	ASM 151710 (TYPE)	GCA_001637525.1	99.72	87.59	33.4
*Bacillus mojavensis*	KCTC 370601 (TYPE)	GCA_000507105.1	99.65	87.51	33.2
*Bacillus vallismortis*	DSM 11031 (TYPE)	GCA_004116955.1	99.65	91.69	45.2
*Bacillus nakamurai*	NRRL B-41091 (TYPE)	GCA_001584325.1	99.65	77.47	20.6
*Bacillus velezensis*	NRRL B-41580 (TYPE)	GCA_001461825.1	99.63	77.11	20.4
*Bacillus amyloliquefaciens*	DSM 7 (TYPE)	GCA_000196735.1	99.36	77.13	20.7
*Bacillus siamensis*	KCTC 13613 (TYPE)	GCA_000262045.1	99.36	77.22	20.7
*Bacillus atrophaeus*	NRRL NRS 213 (TYPE)	GCA_001584335.1	99.29	79.81	22.4
*Bacillus glycinifermentans*	GO-13 (TYPE)	GCA_001042475.2	98.80	73.11	18.9
*Bacillus paralicheniformis*	KJ-16 (TYPE)	GCA_001042485.2	98.73	72.79	18.4

**Table 2:** Biosynthetic gene clusters (BGCs) found in *Bacillus cabrialesii* subsp. *cabrialesii* TE5 during genome mining through the antiSMASH web server.

Region	From	To	BGC Type	Most Similar Known Cluster	Similarity (%)
1.1	11,828	33,438	Sactipeptide	Subtilosin A	100
5.1	300,996	352,779	NRP-metallophore, NRPS	Bacillibactin	100
15.1	1	27,611	NRPS, betalactone	Fengycin	80
15.2	108,515	223,182	TransAT-PKS, NRPS, T3PKS, PKS-like	Bacillaene	100
17.1	162,207	197,906	Other	Bacilysin	100
24.1	217,682	283,063	NPR lipopeptide	Surfactin	86
25.2	161,568	189,160	Phosphonate	Rhizocticin A	100

## Data Availability

The complete genome sequence has been deposited in DDBJ/ENA/GenBank under accession number JBEJUL000000000, BioProject number PRJNA1123860, and BioSample number SAMN41829245. Raw data are available under accession number PRJNA1123860 from the following link: https://dataview.ncbi.nlm.nih.gov/object/PRJNA1123860 (accessed on 15 December 2024).

## Abbreviations

The following abbreviations are used in this manuscript:

AntiSMASH	Antibiotics and secondary metabolite analysis shell
ANI	Average nucleotide identity
BGC	Biosynthetic gene clusters
BLAST	Basic local alignment search tool
CDPS	tRNA-dependent cyclodipeptide synthetases
CDS	Coding DNA sequence
CFE	Cell-free extract
CFU	Colony-forming unit
COLMENA	Colección de microorganismos endófitos y edáficos nativos
DNA	Deoxyribonucleic acid
GBDP	Genome BLAST distance phylogeny
GGDC	Genome-to-genome distance calculator
NB	Nutrient broth
NRP-Metallophore	Non-ribosomal peptide metallophore
NRPS	Non-ribosomal peptide synthetase
OD	Optical density
OGRIs	Overall genome relatedness indexes
PDA	Potato dextrose agar
PDB	Potato dextrose broth
PKS	Polyketide synthetase
RAST	Rapid Annotation Using Subsystems Technology
rRNA	Ribosomal ribonucleic acid
T3PKS	Type III PKS
TYGS	Type Strain Genome Server
